# Development and Characterization of EST-SSR Markers from *Scapharca broughtonii* and Their Transferability in *Scapharca subcrenata* and *Tegillarca granosa*

**DOI:** 10.3390/molecules170910716

**Published:** 2012-09-07

**Authors:** Meng Li, Ling Zhu, Chun-Ya Zhou, Lin Lin, Yan-Jun Fan, Zhi-Meng Zhuang

**Affiliations:** 1 College of Fisheries and Life Science, Dalian Ocean University, Dalian 116023, China; Email: limengzdk@163.com; 2 Key Laboratory of Sustainable Development of Marine Fisheries, Ministry of Agriculture, Yellow Sea Fisheries Research Institute, Chinese Academy of Fishery Sciences, Qingdao 266071, China; Email: chunyat@163.com (C.-Y.Z.); linlinbio@163.com (L.L.); yanjunfan2008@163.com (Y.-J.F.); zhuangzm@ysfri.ac.cn (Z.-M.Z.); 3 College of Fisheries and Life Science, Shanghai Ocean University, Shanghai 201306, China

**Keywords:** ark shell, *Scapharca broughtonii*, EST-SSR markers, transferability

## Abstract

Twenty-five novel EST-derived simple sequence repeat (EST-SSR) markers were developed in the ark shell *Scapharca broughtonii*. Polymorphisms of these EST-SSR markers were evaluated in 48 wild individuals collected from Shidao, Shandong Province, China. A total of 202 alleles were detected at 25 loci. The numbers of alleles per locus ranged from 4 to 14, with an average of 8.08. The observed and expected heterozygosities varied from 0.2917 to 1.000 and from 0.3570 to 0.9002, respectively. After sequential Bonferroni correction for multiple tests, only one locus was found to deviate from Hardy-Weinberg equilibrium. Twenty-five EST-SSR markers showed a high rate of across-species transferability (100%) in *Scapharca subcrenata* and a low rate of across-genus transferability (20%) in *Tegillarca granosa*. These EST-SSRs will be helpful for QTL mapping, molecular breeding and investigation of population genetic diversity in ark shell *S. broughtonii* and other Scapharca species.

## 1. Introduction

The ark shell *Scapharca broughtonii* (*S. broughtonii*), a member of the family Arcidae distributed along the northwestern Pacific coast, is a commercially important bivalve species in Asian countries [1]. However, the natural resources of *S. broughtonii* in China have declined drastically due to the unsustainable exploitation and environmental impacts over the last decades [1]. Characterizing the population structure of *S. broughtonii* would help understanding the effect of overexploitation and environmental changes and provide important information for maintenance and management of ark shell resources.

To date, DNA-based molecular markers, including RFLP [2], RAPD [3], AFLP [4], SSR [5], ISSR [6], mtDNA [7] and SNP [8], have been used extensively in genetic studies. Compared with other types of molecular markers, SSR markers have many advantages including high abundance, random distribution in the entire genome, high information content, codominant inheritance and reproducibility [9]. Generally, SSRs are divided in two categories: genomic SSRs derived from random genomic sequences and EST-SSRs derived from expressed sequence tags. Compared with the development of EST-SSR, genomic SSRs from the repeat-enriched genomic library is very time-consuming, cost-expensive and labor-intensive [10]. Furthermore, Genomic SSRs have neither genic function nor close linkage to transcriptional regions, while EST-SSRs are potentially tightly linked with functional genes that perhaps control certain important genetic characters [9]. In addition, EST-SSR markers contain high transferability because EST-SSRs are derived from expressed sequences that are more conserved than the non-genic sequences and easily found in other relative species [11]. Therefore, more and more EST-SSR markers have been identified and used extensively for comparative mapping [12], DNA fingerprinting [13], biodiversity [13], evolutionary studies [14] and so on.

In the present study, we report the development and characterization of 25 EST-SSRs, and the transferability of EST-SSRs across-species in *Scapharca subcrenata* and across-genus in *Tegillarca granosa*. These EST-SSRs will be helpful for QTL mapping, molecular breeding and investigation of population genetic diversity in ark shell *S. broughtonii* and other *Scapharca* species.

## 2. Results and Discussion

A total of 3005 EST sequences were received by T3 random sequencing. Of these sequences, 84 SSRs (2.8%) were identified [15]. Among the 84 EST-SSRs, 67 (79.8%) were dinucleotide repeats, 14 (16.7%) were trinucleotide, two (2.3%) were tetranucleotide, and one (1.2%) was a hexanucleotide repeat. The most common dinucleotide and trinucleotide repeats were the motif (TA)n (13.4%) and (CAA)n (2.0%) in obtained EST-SSRs. (AT)n (AC)n (CA)n and (GA)n in total accounted for 43.7% of all SSR repeats found in the 84 SSRs [16].

Primer pairs were designed in 72 SSR-containing ESTs. After optimization, the 41 primer pairs (57%) amplified the expected products. Of the 41 primer pairs, 25 (61.0%) were polymorphic, while the others (39%) were monomorphic. The primer sequences and PCR conditions for the 25 polymorphic loci were presented in Table 1. SSR markers derived from ESTs are considered less polymorphic than that of genomic SSR markers [17,18].

**Table 1:** Characteristics of 25 microsatellite loci in 48 *S. broughtonii* individuals.

Locus	Genebank	Repeat	*P*-value	*H*_e_	*H*_O_	PRIMER	Size	Tm	Allele number	*Scapharca subcrenata*	*Tegillarca granosa*
	accession No.	Motif						°C			
KH1			1	0.5923	1	GGGCACTGGACATTTACACAT	362	55			−
	JX105439	(TA)9				CAGTTGTTGTTGGCTGTAGAGAA			5	+	
KH3			0.2542	0.8344	0.8542	ATAGAGCTTTCCCCAGTCCAA	228	55			−
	JX105440	(AC)15				GAACTTGTATATGGTTATGCCACG			11	+	
KH4 *			0.0214	0.6519	0.4889	CTGGTGGACATGCTGTTATTTC	231	55			+
	JX105441	(GT)9				CTGTGAGTGATAGAAAACCCCA			4	+	
KH6			0.0059	0.8884	0.7917	AGTGGAGGGGAGTGTAGTGAAG	297	57			+
	JX105442	(CT)12				GTGATTTTCTTCTGGTTTTCCG			13	+	
KH9			0.6919	0.4557	0.4583	GAGATCAAATGGCACACAAACA	318	55			−
	JX105443	(CA)9				TGCATAGATCCAGTTCTGCCTT			8	+	
KH12			0.0263	0.357	0.2917	CAGAACGATTTACTGGCCTTTT	358	55			+
	JX105444	(AC)9				CGGACCTCAAACAACTTACTTTC			4	+	
KH19			0.066	0.7779	0.7021	TGCGGTTAGTTGAATTTGTCAG	220	60			+
	JX105445	(GC)8				TATACTCGGGAGAAGGGTAACG			12	+	
KH20			0.0539	0.7822	0.6667	TGCCTGCAAATAAAGGACACTA	352	50			−
	JX105446	(GA)11				TTTACAAACCCCTCTTTCTCTCC			6	+	
KH24 *			0.0094	0.75	0.6042	TGCAGTACATTAACAGGCCAAT	260	50			−
	JX105447	(TGT)7				AAGTAATCTCCAAAGTCGTGGC			6	+	
KH33			0.945	0.8379	0.9167	TCCTTATGACAATCCACTTCACAC	313	50			−
	JX105448	(TACA)6				GCGGCTATCCTGAAACTAAAAG			10	+	
KH36 *			0.0079	0.5215	0.3542	TTCAACAGAACAACCTGTGACC	337	50			+
	JX105449	(TC)7				GCACCACAAAATAGACCAACAA			5	+	
KH40			0.3395	0.8217	0.8125	CGTATCCATATCCGTGTTGATT	340	50			−
	JX105450	(GA)7				CATGTGTTGGGGTTTTCAGTAT			10	+	
KH46			1	0.673	1	CACAACAAAGGGTCTGGTTTATG	247	50			−
	JX105451	(CAA)7				CGTCAAAATGGGAAAATCTGTC			6	+	
KH48			1	0.6472	0.7708	AACAGTGAAATGAGCAGTGGAA	370	50			−
	JX105452	(ACG)7				CGGTTACGGAAGGAATTTGTAA			10	+	
KH52 *^△^			0	0.8445	0.8912	TTTTCGTTTAATTTTCTGTTG	281	55			−
	JX105453	(AT)6				GATTCAGGTTGTCAGGGA			10	+	
KH55			0.9247	0.8912	0.9583	CAAAGTAGCGCCAGATACAGAA	370	50			−
	JX105454	(CT)7				CGAATACGAGAAGGTCTGCAC			13	+	
KH56			0.0238	0.9002	0.7917	CACCTGCCCTAAGAAAAT CGTACGTCGAAATAGTCAT	248	50			−
	JX105455	(CA)20							14	+	
KH57			0.9789	0.7547	0.8726	TTTATTCTTAACACTGGCAA	289	50			−
	JX105456	(CA)10				TTAGGCATGAATGGGAAA			7	+	
KH58			0.0034	0.5866	0.5531	GATTCGGATACCAAGGGACATA	338	52			−
	JX105457	(AT)8				GCATTTTCCATCACAGAACTGA			7	+	
KH59 *			0.1215	0.7246	0.5625	GCTGTGGTTGGATGGGTT TACCCTGTCTTAGAATGTCAGCA	326	52			−
	JX105458	(ATT)6							8	+	
						ACGAGAAGAAGAGGTGAT					
KH61	JX105459	(CAT)8	0.3273	0.7689	0.75	TAAAACTGAATGGTGATG	351	50	8	+	−
KH63			0.9258	0.605	0.6875	CTATTCATGGACACCAGATGC	317	55			−
	JX105460	(TA)6				CCGAAAACCAAAAGACATACAC			5	+	
KH65			1	0.7057	0.9792	TAATTGAAAACCATCCAGGG AACCACATCACAGTCAAACAGA	219	48			−
	JX105461	(TG)7							5	+	
KH67			1	0.8007	1	ATTATCGTCTATTGCTGCCGAG	182	45			−
	JX105462	(CA)7				TCTAGTCTCCCCTGAACCAGAA			11	+	
KH71			1	0.6945	1	ACATGTACCTTGAATAAC	246	55			−
	JX105463	(CA)7				GTATGCTTCAGTGTATCT			4	+	

*P*-value, Hardy-Weinberg probability test; Tm, optimized annealing temperature; *H*_o_, observed heterozygosity; *H*_e_, expected heterozygosity; ^△^ indicated deviation from Hardy-Weinberg equilibrium (*P*-value < 0.002); * indicated harboring null alleles (null allele frequency >5%); + stands for amplified successful; − stands for amplified unsuccessful.

However, our study showed that the ratio of polymorphic loci from EST-SSRs was similar to those of from genomic-SSRs in *S. broughtonii*, where the ratios of polymorphic loci were 64.7% [19] and 40% [20], respectively. Similar observations were also found in the *Crassostrea virginica* [21,22,23]. The ratios of polymorphic loci from EST-SSRs were 80% [21], while those from genomic-SSRs in *C. virginica* were 70% [22] and 85.8% [23], respectively.

The 25 polymorphic loci were further characterized in a population of 48 individuals. The number of alleles per locus ranged from 4 to 14, with an average of 8.08, which was low in comparison with those of genomic-SSRs in *S. broughtonii*, which were 11.5 [1], 13 [19] and 17.4 [20], respectively. In contrast, the number of average alleles from EST-SSRs (10.11) [21] are higher than those of genomic SSRs (5.4 and 5.7) in *C. virginica* [22,23]. Different results may have been caused by different populations and sample sizes used in different studies [21]. The observed and expected heterozygosities ranged from 0.2917 to 1.000 and from 0.3570 to 0.9002, with the average of 0.7503 and 0.7147, respectively. After sequential Bonferroni correction for multiple tests, only one locus deviated from Hardy-Weinberg (*p* < 0.002). In previous study, the numbers of loci from genomic-SSRs deviated from Hardy-Weinberg equilibrium in *S. broughtonii* were two out of 11 (18.2%), four out of 10 (40%) and nine out of 12 (75%), respectively, which were higher than that of EST-SSRs (4%) in our research [1,19,20]. Different populations and sample sizes used in studies may have led to these different results [21]. Gene linkage can’t be found in all of the loci. Of the 25 loci, KH4, KH24, KH36, KH52 and KH59 showed signs of null alleles.

Because EST-SSRs are more conserved than the non-genic sequences, EST-SSR markers share high transferability [11]. After cross-species detection, all of the 25 reliable SSRs were amplified successfully and showed polymorphism in *S. subcrenata*. In cross-genus application, only five of 25 loci (20%) were amplified stably and detected polymorphism in *T. granosa*. This was the first report about the cross-genus amplification of EST-SSRs in *S. broughtonii*. With the EST-SSR markers developed in our study, further studies can be conducted for comparative mapping [12], DNA fingerprinting [13], biodiversity [13], and evolutionary studies [14] and so on. These will help advance the investigation of the genetic population structure and genetic diversity in this species.

## 3. Experimental

### 3.1. DNA Extraction from S. broughtonii, S. subcrenata and T. granosa

Forty-eight wild individuals of *S. broughtonii*, six of *S. subcrenata* and six of *T. granosa*, respectively, were collected from Shidao, Shandong Province, China. Genomic DNA was extracted from adductor muscle by standard proteinase K digestion, phenol-chloroform extraction, and DNA precipitation [3]. 

### 3.2. cDNA Library Construction and EST-SSR Identification

A cDNA library was constructed from the whole of ark shell *S. broughtonii*, using the ZAP-cDNA synthesis kit and ZAP-cDNA Gigapack III Gold cloning kit (Stratagene, La Jolla, CA, USA). Random sequencing of the library using T3 primer yielded 3005 successful EST sequences. These ESTs were screened to find regions containing SSRs using SSRHUNTER program, where the parameters were set for detection of di-, tri-, tetra-, penta- and hexa-nucleotides motifs with a minimum of six repeats. A total o f 84 SSR loci were identified and 72 were appropriate for SSR marker optimization.

### 3.3. Primer Design and Validation

Primers flanking microsatellite were designed using the PRIMER PREMIER 5.0 program (PREMIER Biosoft International, Palo Alto, CA, USA). The major parameters for primer design were set as follows: primer length from 18 to 24 nucleotides, PCR product size from 180 to 360 bp, optimum annealing temperature at 55–65 °C, and GC contents from 40% to 60%.

Primer pairs were validated using six ark shell individuals by PCR and denaturing PAGE. PCR was performed on a Veriti Thermal Cycler (Applied Biosystems, Carlsbad, CA, USA) in a 25 μL reaction volume, concluding 2.5 μL of 10 × PCR buffer, 1.5 μL of MgCl_2_ (25 mmol L^−1^), 2.0 μL of dNTP (2.5 mmol L^−1^), 1 μL of each primer (10 μmol L^−1^), 15.8 μL of PCR-grade water, 0.2 μL (1 U) of Taq polymerase (Promega, Madison, WI, USA), and 1 μL of 10–100 ng DNA. The PCR temperature profile was 94 °C for 5 min, followed by 33 cycles of 95 °C for 45 s, 45 s at the locus-specific annealing temperature (Table 1), 72 °C for 45 s, and the final extension step at 72 °C for 10 min. The PCR products were separated on 6% denaturing polyacrylamide gel, and visualized by silver-staining. The size of allele was estimated according to the pBR322/MspI marker (TianGen, Beijing, China).

### 3.4. EST-SSR Polymorphism Analysis and Transferability in S. Subcrenata and T. Granosa

Loci with stable, specific and polymorphic PCR fragments were characterized in 48 ark shells from a wild population, as well in *S. subcrenata* and *T. granosa* to assess their transferability. PCR and denaturing PAGE were performed at the same condition as above. The observed and expected heterozygosities together with tests for Hardy-Weinberg equilibrium and linkage disequilibrium were calculated by GENEPOP 4.0 [15]. Null allele frequencies were analysed by MICRO-CHECKER 2.2.3 [16]. All results for multiple tests were corrected using sequential Bonferroni’s correction.

## 4. Conclusions

Here, we report the development and characterization of a set of EST-SSRs, which was derived from a cDNA library of ark shell *S. broughtonii* by random sequencing. Twenty-five polymorphic EST-SSR markers not only are suitable for evaluating the population genetics of ark shell, but also showed a high rate of across-species transferability in *S. subcrenata* and a low rate of across-genus transferability in *Tegillarca granosa*. These EST-SSRs will be helpful for QTL mapping, molecular breeding and investigation of population genetic diversity in ark shell *S. broughtonii* and other Scapharca species.
